# Improving maternal health services through social accountability interventions in Nepal: an analytical review of existing literature

**DOI:** 10.1186/s40985-020-00147-0

**Published:** 2020-12-21

**Authors:** Adweeti Nepal, Santa Kumar Dangol, Anke van der Kwaak

**Affiliations:** 1 Save the Children, Surkhet, Karnali Province Nepal; 2 CARE International, Nepal, P.O Box 1611, 4/288- SAMATA Bhawan-Dhobighat, Lalitpur, Nepal; 3 Royal Tropical Institute (KIT), Mauritskade 64, 1092 AD Amsterdam, The Netherlands

**Keywords:** Social accountability, Community participation, Maternal health services, Health system, Responsiveness, Quality of care

## Abstract

**Background:**

The persistent quality gap in maternal health services in Nepal has resulted in poor maternal health outcomes. Accordingly, the Government of Nepal (GoN) has placed emphasis on responsive and accountable maternal health services and initiated social accountability interventions as a strategical approach simultaneously. This review critically explores the social accountability interventions in maternal health services in Nepal and its outcomes by analyzing existing evidence to contribute to the informed policy formulation process.

**Methods:**

A literature review and desk study undertaken between December 2018 and May 2019. An adapted framework of social accountability by Lodenstein et al. was used for critical analysis of the existing literature between January 2000 and May 2019 from Nepal and other low-and-middle-income countries (LMICs) that have similar operational context to Nepal. The literature was searched and extracted from database such as PubMed and ScienceDirect, and web search engines such as Google Scholar using defined keywords.

**Results:**

The study found various social accountability interventions that have been initiated by GoN and external development partners in maternal health services in Nepal. Evidence from Nepal and other LMICs showed that the social accountability interventions improved the quality of maternal health services by improving health system responsiveness, enhancing community ownership, addressing inequalities and enabling the community to influence the policy decision-making process. Strong gender norms, caste-hierarchy system, socio-political and economic context and weak enforceability mechanism in the health system are found to be the major contextual factors influencing community engagement in social accountability interventions in Nepal.

**Conclusions:**

Social accountability interventions have potential to improve the quality of maternal health services in Nepal. The critical factor for successful outcomes in maternal health services is quality implementation of interventions. Similarly, continuous effort is needed from policymakers to strengthen monitoring and regulatory mechanism of the health system and decentralization process, to improve access to the information and to establish proper complaints and feedback system from the community to ensure the effectiveness and sustainability of the interventions. Furthermore, more study needs to be conducted to evaluate the impact of the existing social accountability interventions in improving maternal health services in Nepal.

## Background

The Government of Nepal (GoN) launched the Safe Motherhood Programme in 1997 to improve maternal and neonatal outcomes [1]. The programme particularly placed emphasis on addressing financial barriers to access maternal and newborn health services, institutional delivery and skilled birth attendant (SBA) at each birth [2, 3]. As a result, institutional delivery is widely promoted which led to nationwide expansion of birthing centres. Since then, there has been a considerable increment in the coverage of institutional delivery, from 8% in 1996 to 57% in 2016. However, expansion of birthing centres has not ensured the quality of care (QoC) at service delivery point as envisioned by the programme [4]. Despite the intense effort of the programme, Nepal is still facing challenges to reduce maternal mortality rate (MMR) as per the national and global target; the statistics is 239 per 100,000 live births [5]. Two major causes—persistent equity and quality gap—are observed as underlying causes for the slow and steady progress in maternal health outcomes in Nepal [2, 6]. The access and utilization of maternal health services are still primarily low among less educated women, women of the lowest wealth quintile and hard-to-reach (Table 1) [5].

**Table 1:** Overview of maternal health service utilization in Nepal by background characteristics

Maternal health services	Total coverage (%)	Wealth quintile		Education		Residence	
		Highest	Lowest	Above secondary level	No education	Urban	Rural
Antenatal care coverage	83.6%	95.5%	73.8%	94.5%	73.3%	87.0%	79.5%
Institutional delivery	57.4%	89.6%	33.9%	85.4%	36.4%	68.6%	44.2%
Delivery assisted by skilled provider	58.0%	88.7%	33.9%	84.9%	37.6%	67.7%	46.8%
Postnatal care coverage	56.7%	81.2%	36.7%	80.2%	41.7%	63.9%	48.4%

Source: NDHS 2016 [5]

A national survey of birthing centres reported only 5% of birthing centres satisfy the requirements of the Safe Motherhood Programme. Only half of the nursing staff of birthing centres are certified SBA. The infection prevention practices are considerably low among the staffs [4]. The health system-related factors such as cleanliness, hygiene, poor counselling, audio-visual privacy, lack of essential drugs and supplies, staff absenteeism and their attitude are major reasons behind dissatisfaction to maternal health services among the maternity clients [7]. Similarly, the lack of confidence in services being provided is a reason for underutilization of primary level of care which has resulted into overcrowding at the maternity unit in referral hospitals [8]. Available evidences, particularly from the developing contexts, suggest that it is essential not only to go beyond maximizing essential interventions to accelerate the progress in maternal health outcomes but also to invest for the quality maternal health services through accountable health system [9, 10].

Social accountability refers to an approach that enables the citizens to make service providers and policymakers responsible and answerable for their acts [11]. It has been considered an effective strategy to aggregate the voice of disadvantaged and marginalized people, enhance citizen-service provider relationships and improve state/service provider responsiveness towards effective and quality service delivery contributing to better maternal health outcomes in developing context [11–14].

The GoN with its commitment to inclusive and quality health care services has explicitly emphasized accountability and governance in its health strategy as a core principle to attain sustainable development goals (SDGs) in relation to maternal and newborn health [2, 15]. Major focus has been on universal health coverage (UHC) and its progressive expansion with continuous improvement in the quality of care being delivered, making service more affordable and covering wider population in need, especially the poor and vulnerable through social accountability interventions [2, 6].

Social accountability interventions aimed to improve maternal health are reported in many developing countries, but such reports in Nepal are very limited. Through the review of available literature, this paper intends to address the knowledge gap. The finding of the study is expected to provide comprehensive information about social accountability initiatives and right-based approaches to policymakers and health service providers engaged in health system, strengthening social accountability initiatives and right-based approaches to improve quality of maternal health services in Nepal.

## Methods

**Fig. 1:**
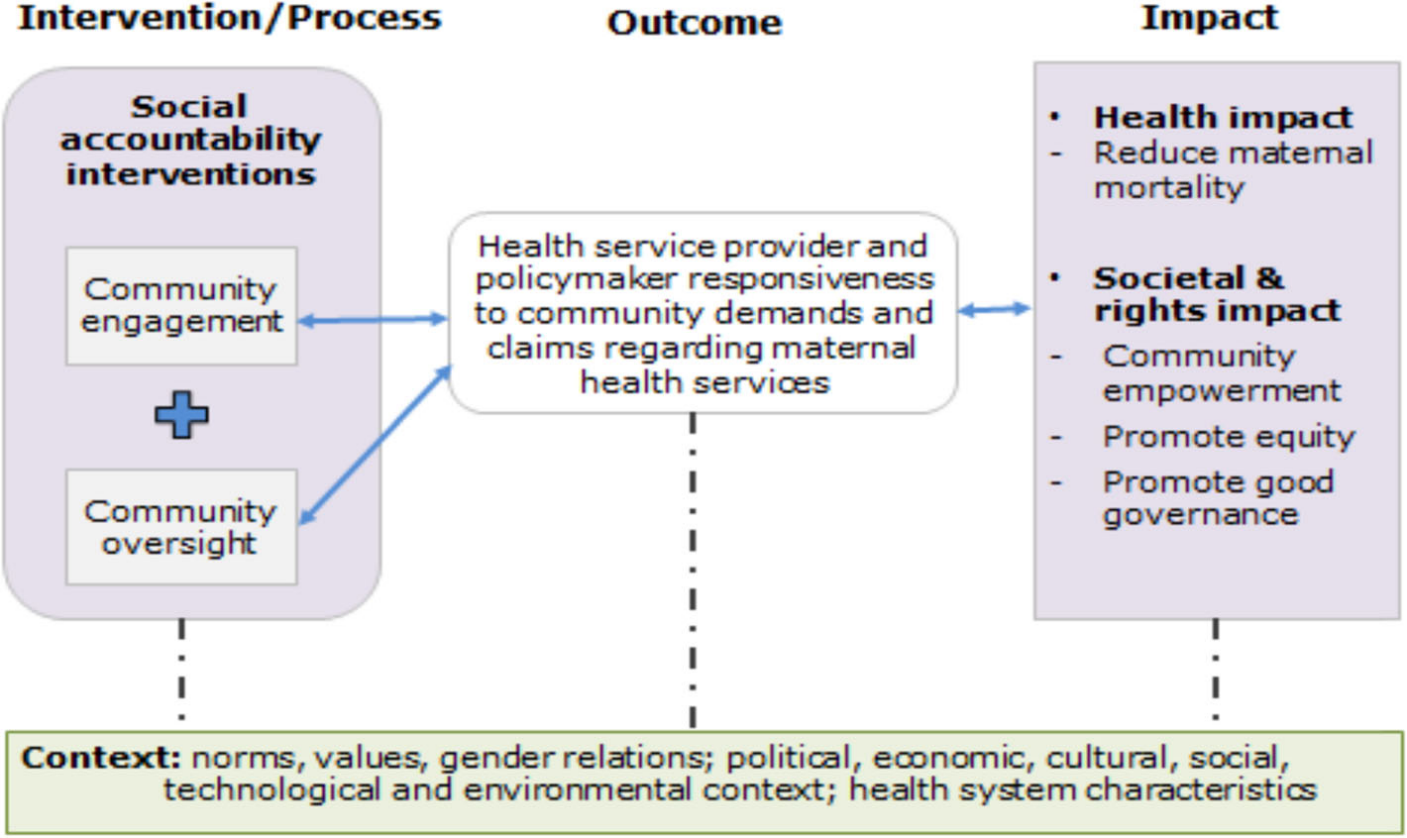
Conceptual framework of social accountability adapted from Lodenstein et al. [17]

In-depth review of literature from different LMICs including Nepal was conducted to explore the social accountability interventions and their outcome in maternal health services. The review was done in December 2018 to May 2019. The adapted framework (Fig. 1) of Lodenstein et al. was used to critically analyse and synthesize existing literature. The framework was chosen over the World Bank accountability framework as the World Bank’s model specifically focuses on the citizen-policymaker relationship and does not discuss the importance of the contextual factors [16].

Originally, Lodenstein et al. conceptualized the social accountability interventions as a citizen-driven mechanism, where citizens voice their concern on the quality of services or/and service providers’ and policymakers’ performance. In return, the service providers and policymakers are asked to respond to those concerns [17]. Equally, citizen oversight the service providers’ and policymakers’ actions and decisions using accountability mechanism which result in improved responsiveness of service providers and policymakers. Improved responsiveness ultimately creates health and rights impact. The interventions, outcome and impact are, however, influenced by different contextual factors [17].

World Health Organization (WHO) has recognized community engagement interventions as a cornerstone to improve the quality of maternal and newborn care. Accordingly, Nepal has adopted a community participation approach in its health system and has incorporated community engagement and community oversight as central elements of the social accountability interventions [2, 18]. In this review, the term community engagement refers to the community voice or expression of concerns for the quality maternal health services without formal enforcement and community oversight which includes joint monitoring and evaluation of the maternal health services and health system. The interventions are anticipated to improve health service providers’ and policymakers’ responsiveness toward delivering quality maternal health services. Improved responsiveness ultimately creates health and rights impact by reducing maternal mortality and promoting equity and good governance.

The main keywords related to conceptual framework, that is, ‘social accountability interventions,’ ‘maternal health services,’ ‘community participation,’ ‘outcomes’ and ‘Nepal’ were combined using Boolean operators ‘OR’ and ‘AND’ to narrow down the search and produce conclusive evidence on the interventions. Literature was searched and extracted via search engines such as Google and PubMed database (search strategy presented in Appendix 1). For the national reports and data, authentic websites of GoN, different development agencies and organizations websites which include Ministry of Health and Population (MoHP) Nepal, Department of Health Services (DoHS) Nepal, Ministry of Federal Affairs and Local Development (MoFALD), WHO, World Bank and CARE Nepal were visited. Additionally, a snowball approach was used to access references mentioned in key review papers. While limited evidence related to outcomes of social accountability interventions in Nepal were found, evidences from other LMICs having similar maternal health and socio-economic context were included.

**Fig. 2:**
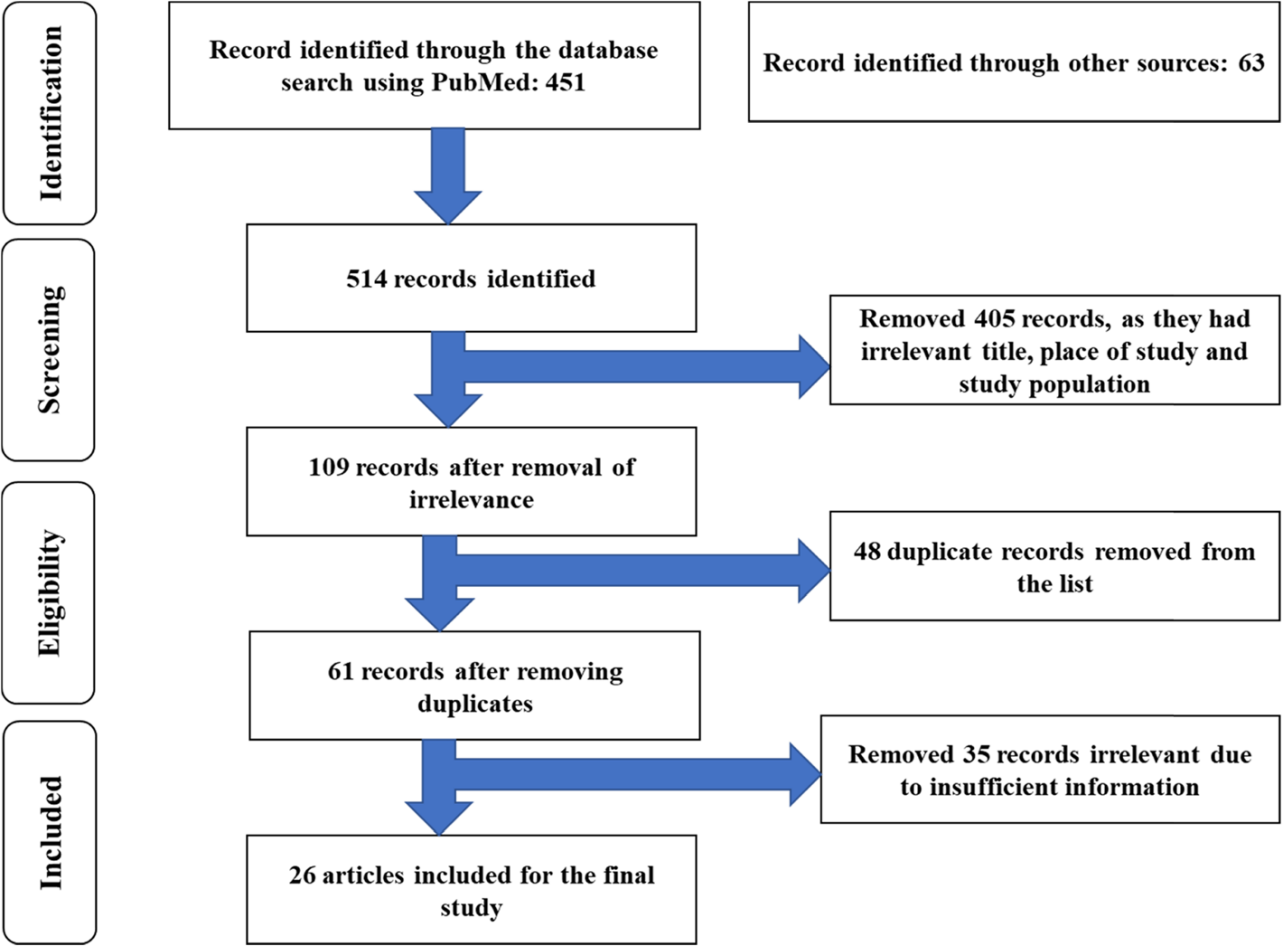
Article selection process

Aligning with the Local Self-Governance Act 1999, the MoHP implemented decentralization in the health sector in 2000, the same year was set as the lower limit for literature search. All published as well as unpublished (grey) and open-access literature between January 2000 and May 2019 were included in this review. Some relevant policy documents that were originally published in the Nepali language and later translated into English are also included in the review. Studies focussing on other approaches and interventions to improve maternal health services were excluded. For the national data, researchers NDHS 2016 and Annual report of the DoHS were used as a reference. Altogether, 451 free full-text articles from January 2000 to May 2019 were identified from the PubMed. Additional 63 articles were identified from Google Scholar, ScienceDirect and reference section of selected articles. Among the total 514 articles, 405 were removed from the review list as they did not match the title of study, place of the study and study population. From the remaining 109 articles, 48 duplicate articles were excluded. Similarly, 35 articles were removed as they did not contain relevant information, and 26 articles alongside national reports and documents were finally reviewed. The article selection process is presented in Fig. 2. The details of the articles included in the review with their key characteristics are presented in Table 2 (see Appendix 2 for the details of all literature included).

**Table 2:** Summary of articles in this review

SN	Author(s)	Title	Year of publication	Study design	Indicator(s) included
1	Tunçalp Ӧ, Were W, MacLennan C, Oladapo O, Gülmezoglu A, Bahl R, et al.	Quality of care for pregnant women and newborns – the WHO vision	2015	Commentary	WHO quality of care framework
2	Hulton L, Matthews Z, Bandali S, Izge A, Daroda R, Stones W	Accountability for quality of care: Monitoring all aspects of quality across a framework adapted for action	2016	Analytical review	Elements of WHO’s quality of care framework
3	Lodenstein E, Dieleman M, Gerretsen B, Broerse JE	Health provider responsiveness to social accountability initiatives in low- and middle-income countries: a realist review	2017	Realist review	Mechanism of social accountability initiatives
4	Mafuta EM, Dieleman MA, Hogema LM, Khomba PN, Zioko FM, Kayembe PK, et al.	Social accountability for maternal health services in Muanda and Bolenge Health Zones, Democratic Republic of Congo: a situation analysis	2015	Exploratory study	Existing mechanisms regarding social accountability in maternal health services
5	Lodenstein E, Dieleman M, Gerretsen B, Broerse JE.	A realist synthesis of the effect of social accountability interventions on health service providers’ and policymakers’ responsiveness	2013	Systematic review	Elements of social accountability initiatives
					Context
					Mechanism
					Outcome
6	van den Broek N, Graham W.	Quality of care for maternal and newborn health: the neglected agenda	2009	Commentary	Maternal Perinatal Death Reviews
					Criterion-based audit
7	Bandali S, Thomas C, Hukin E, Matthews Z, Mathai M, Ramachandran Dilip T, et al.	Maternal Death Surveillance and Response Systems in driving accountability and influencing change	2016	Review	Maternal mortality
8	Biswas A, Rahman F, Halim A, Eriksson C, Dalal K	Maternal and Neonatal Death Review (MNDR): A Useful Approach to Identifying Appropriate and Effective Maternal and Neonatal Health Initiatives in Bangladesh	2014	Quantitative and qualitative	Accountability in maternal and neonatal health
9	Gullo S, Galavotti C, Sebert Kuhlmann A, Msiska T, Hastings P, Marti CN.	Effects of a social accountability approach, CARE’s Community Score Card, on reproductive health-related outcomes in Malawi: A cluster-randomized controlled evaluation	2017	Cluster-randomized controlled evaluation	Service utilization
					Perceived service quality
					Health behaviour
					Supportive care outcomes
10	Hamal M, Heiter K, Schoenmakers L, Smid M, de Cock Buning T, De Brouwere V, et al.	Social Accountability in maternal health services in the far-western development region in Nepal: An exploratory study	2019	Exploratory Study	Governance
					Maternal health services
11	Gurung G, Gauld R, Hill PC, Derrett S.	Citizen’s Charter in a primary health-care setting of Nepal: An accountability tool or a “mere wall poster”?	2018	Case Study: a quantitative survey	Health governance
12	Atela M, Bakibinga P, Ettarh R, Kyobutungi C, Cohn S.	Strengthening health system governance using health facility service charters: a mixed methods assessment of community experiences and perceptions in a district in Kenya	2015	Mixed method study	Health governance
13	Gurung G, Derrett S, Gauld R, Hill PC.	Why service users do not complain or have ‘voice’: a mixed-methods study from Nepal’s rural primary health care system	2017	Mixed method study	Voice mechanism
14	Oostdam S, Hamal M, Dieleman M, De Brouwere V, Bardají A, Tiwari DP, et al.	Social accountability in maternal health services in Baglung district, Nepal: a qualitative study	2018	Qualitative study	Voice mechanism
15	Shakya HS, Adhikari S, Gurung G, Pant S, Aryal S, Singh AB, et al.	Strengthening national health systems for improving efficiency of health service delivery in Nepal	2012	Literature review	Health governance in maternal and child health services
16	Khatri RB, Mishra SR, Khanal V.	Female Community Health Volunteers in Community-Based Health Programs of Nepal: Future Perspective	2017	Perspective	Community engagement
17	Hamal M, Dieleman M, De Brouwere V, de Cock Buning T.	How do accountability problems lead to maternal health inequities? A review of qualitative literature from Indian public sector	2018	Scoping review	Health governance in maternal health services
18	Manandhar D, Osrin D, Shrestha B, Mesko N, Morrison J, Tumabhangphe K, et al.	Effect of a participatory intervention with women’s group on birth outcomes in Nepal: cluster randomized controlled trial	2004	Cluster-randomized controlled trail	Women's participation
					Maternal mortality
					Neonatal mortality
					Quality of Care
19	Prost A, Colbourn T, Seward N, Azad K, Coomarasamy A, Copas A, et al.	Women’s groups practising participatory learning and action to improve maternal and newborn health in low-resource settings: a systematic review and meta-analysis	2013	Meta-analysis	Women's participation
					Maternal mortality
					Neonatal mortality
					Quality of Care
20	Rifkin SB.	Examining the links between community participation and health outcomes: a review of the literature	2014	Literature review	Community participation
21	Paudel NR.	Inclusive Governance: A Case Study of Civil Service in Nepal	2016	Exploratory Study	Governance
					Bureaucracy
22	Gurung G, Derrett S, Hill PC, Gauld R.	Nepal’s Health Facility Operation and Management Committees: exploring community participation and influence in the Dang district’s primary care clinics	2018	Qualitative study	Community participation
					Community oversight
					Health governance
23	McCoy DC, Hall JA, Ridge M.	A systematic review of the literature for evidence on health facility committees in low- and middle-income countries	2012	Systematic review	Health governance
					Contextual factors
24	Gurung G, Derrett S, Hill PC, Gauld R.	Governance challenges in the Nepalese primary health care system: time to focus on greater community engagement?	2016	View point	Health governance
					Community engagement
25	Mafuta EM, De Cock Buning T, Lolobi DL, Mayala PM, Mambu TNM, Kayembe PK, et al.	Factors influencing the capacity of women to voice their concerns about maternal health services in the Muanda and Bolenge Health Zones, Democratic Republic of the Congo: a multi-method study	2018	Mixed method study	Voice mechanism
					Health service responsiveness
					Quality of Care
					Maternal mortality
					Contextual factors
26	Hulton L, Matthews Z, Martin-Hilber A, Adanu R, Ferla C, Getachew A, et al.	Using evidence to drive action: A “revolution in accountability” to implement quality care for better maternal and newborn health in Africa	2014	Analytical review	Quality of Care

This review has summarized the findings according to the main elements of the conceptual framework linking it to QoC. The framework is based on theoretical perspectives that include social accountability, social accountability interventions, health system responsiveness and QoC, as defined in Table 3.

**Table 3:** Conceptual definition

**Social accountability:** Social accountability is an approach that enhances accountability through civic engagement. The civic includes citizens and/or civil societies who directly or indirectly hold service providers accountable for their actions. This approach is commonly demand-driven; however, it can be supported by the state as well through the formal mechanism. In the health system, it ensures the quality of care by empowering citizen, protecting their rights, preventing corruption and improving governance [11, 16]. **Social accountability interventions:** In this review, social accountability interventions refer to the approaches, processes, tools, interventions, initiatives that involve community engagement and oversight to enhance health system responsiveness. This definition is adopted from the concept of Lodenstein et al. 2013 [17]. **Health system responsiveness:** Health system responsiveness signifies as an ability of health system (both health services and policymakers) to respond valid expectations of service users and to protect their rights to get adequate and timely care [19]. In this paper, the health system responsiveness refers to the health service providers’ and policymakers’ responses toward community voice and demand regarding quality maternal health services. The responses include a tendency to implement change in their behaviour and attitude, decision-making and management structure for quality services at the point-of-service-delivery which is adapted from Lodenstein et al. 2017 [13]. **Quality of care:** The QoC in maternal health services involves a proper use of effective clinical and non-clinical interventions, strengthen health infrastructure and optimum skill and attitudes of health service providers. Improved QoC results in women and service provider satisfaction and produces better maternal health outcomes [9, 20]. Furthermore, the perception of women, their family and community about the available maternal health services influence their health-seeking behaviour. Therefore, community engagement is the key to improve the quality of maternal health services [9, 10, 17]. In this review, we have adopted following indicators based on National health sector strategy (NHSS) (2015-2020) [6] and the Safe Motherhood Programme [2] to assess the quality of maternal health services. They are (1) Infection prevention practices at health facility/birthing center, (2) Availability of trained human resources at Birthing Center, (3) Attitude of health workers, (4) Availability of equipment and supplies, (5) Coverage of services: ANC, PNC, Institutional deliveries, (6) Client satisfaction, (7) Waiting time to receive the services and (8) Confidentiality.	

## Results

### Social accountability interventions and their outcome in maternal health services in Nepal

In Nepal, different social accountability tools have been used and tested by GoN and development partners to ensure community engagement in social accountability interventions in maternal health services. Similarly, there are established structures in the health system which facilitate community oversight to ensure the governance for improved quality maternal health services and promote social accountability. The major social accountability interventions and contextual factors affecting the interventions are listed in Table 4.

**Table 4:** Overview of social accountability interventions in maternal health services and major contextual factors influencing the interventions

Community engagement tool	Community oversight mechanism	Contextual factor influencing social accountability interventions in Nepal
• Social Audit • Maternal Perinatal Death Surveillance and Response • Community Score Card/Community Health Score Board (CSC/CHSB) • Citizen Charter • Grievance/complain handling tool	• Health Facility Operation and Management Committee (HFOMC) • Female Community Health Volunteers (FCHVs) • Mothers’ Group for Health • Civil Society Organizations (CSOs)	• Socio-Cultural Context ▪ Gender Norms ▪ Social Structure ▪ Awareness, value, beliefs, and practices • Political and economic context • Health System Context ▪ Client-provider relationship ▪ Resource availability ▪ Monitoring and evaluation

The key findings related to the social accountability interventions, process of the interventions and outcomes are presented in Table 5.

**Table 5:** Key findings of the social accountability interventions, its process and outcomes

Community Engagement		
Interventions	Process	Outcomes
i. **Social Audit:** The GoN initiated a social audit in 2009 to ensure the quality implementation of “*Aama programme”*^a^*.* The intervention aimed to increase women’s and community awareness, promote transparency in the decision process of health facility, make the health workers and decision-makers accountable and responsive for quality maternal health services, set a culture of demand for information from the health facility as well as strengthen mutual accountability between service providers and users [20, 21].	The programme is implemented through the local non-government organizations (NGOs) which are identified through a competitive process [20, 22]. The NGOs facilitate the process of social audit where community and local stakeholders are invited to review the performance, identify existing problems and challenges of the health facility as well as develop an action plan for improving quality of services. The process takes 5-6 days to complete in one health facility in the initial stage. However, time implication is less in the follow-up programme [22].	An evaluation study [22] undertaken in health facilities of four districts of Nepal, namely Palpa, Rupandehi, Jhapa and Ilam, found that social audit resulted in the overall improvement in the health service providers’ behaviour, attitude and regularity. Clients and patients received more equitable and dignified treatment. Similarly, antenatal care (ANC) and institutional delivery incentives were timely provided to the beneficiaries. The intervention also improved the dialogue between the community, health service providers and health facility committee. Community concerns were incorporated in the health facility action plan.
ii. **MPDSR:** The GoN initiated the programme in 1990 at the tertiary level hospital with the support of WHO. Since then it has been scaling up at different levels of care at different phases with improvisation from the learnings of the programmes [23]. It is a supply-side intervention initiated to improve quality of maternal health services which is focused on routine identification and notification of maternal and perinatal deaths, determinants of death causes and use of the information to improve quality of care to avoid future deaths [23].	The MPDSR committee is formed at district, hospital and local health facility level. At the local level committee, there is a separate verbal autopsy and cause of death assignment team [23]. The community-based intervention has been implemented in 6 districts whereas facility-based intervention has been expanded in 65 hospitals in 38 districts [2].	No study could be found on the impact of MPDSR on maternal health in Nepal. Hence, the evidence from another similar context is presented. A study in Bangladesh reported that the MDR helped to recognize the causes of maternal deaths in the community and brought the attention of decision-makers to respond and address the issues appropriately. Further, it is observed to result in deploying competent human resource such as medical officer, SBA at birthing centres to manage complications, and ensuring an adequate supply of necessary equipment which ultimately improved the QoC and provider-user satisfaction and finally in the increased uptake of maternal health services and decreased maternal mortality in Bangladesh [24]. A similar finding was reported in Nigeria where MDR played an influential role in improving health service providers’ and policymakers’ responsiveness toward addressing causes of maternal deaths [25].
iii. **CSC/CHSB:** CSC is a tool designed to promote participation, transparency, and accountability among service providers, service users and decision-makers. The tool provides an opportunity for the community to evaluate the quality of services, express their dissatisfaction and voice health rights [26, 27]. The CHSB is an adopted form of CSC. It creates an environment for tripartite dialogue among service providers, beneficiaries and decision-makers which facilitates monitoring and performance of maternal neonatal and child health (MNCH) services [28, 29].	CSC is a process-oriented tool, where community people along with service providers and stakeholders monitor and evaluate health service against agreed indicators. With the indicators, quality of services, health facility performance, and health governance are monitored using the defined scale or score. At the end of the process, community along with service providers develop an action plan and prioritize the activities based on the given score for further improvement [27]. In the CHSB intervention, as in CSC, community people, service providers, and decision-makers jointly discuss the MNCH issues and then provide mutually agreed scores on the performance indicators and develop action plans for further improvement. The action plans are reviewed semi-annually following the same process. It creates a space for providing immediate feedback to service providers and decision-makers and accordingly responses toward raised concerns [21, 28].	The CSC is found effective for promoting direct feedback mechanism and efficient use of resources at a health facility in Nepal [26]. No study found impact of CSC on maternal health in Nepal. The same is observed regarding the impact of CHSB in Nepal. The CHSB is adopted in Nepal after a successful trial in Malawi where the intervention is found to increase interaction between the health service providers, members of the health committee and community; this improved accountability of health worker as well as the quality of antenatal and postnatal care services. Women were treated better at health facilities and the service utilization trend increased. However, the tool has no significant impact on the indicators which needed the attention of higher-level government authorities [30].
iv. **Citizen Charter:** Citizen Charter is an information board displayed at all public service centres. Since 2007, GoN has made it mandatory to have charters installed at clearly visible areas within the public office/facilities [27, 31].	Citizen charter includes the information about the availability of services with cost, essential requirements to access services, name of the contact person, time required and name of the person to redress the grievances if any [21, 27].	A study mapping awareness and factors influencing the implementation of citizen charter in health facility concluded that it promotes the transparency of health facility and accountability of health workers towards service users if well implemented [32]. A similar conclusion was drawn by a study conducted in Kenya [33].
v. **Grievance/Complaint handling tool:** The grievance/complaint handling intervention refers to the provision that allows citizens to file complaint against services and system that hear and address the complaint/grievances [27]. The compliant boxes are major complaint grievance/Complaint handling tool in health system. In some locations, the government has established the digital tool [21].	In the health facility, suggestion or complaint box is a widely used tool for handling the grievances and complaints [21]. The box is placed in the premises of health facilities to receive the complaints and/or grievances from service users, communities as well as other stakeholders about the health services they receive.	No evidence found on the outcome of the grievance/complaint handling tool in improving the responsiveness of the health system.
**Community oversight**		
i. **HFOMC:** The HFOMC is a local level oversight mechanism in the health system which is responsible for the overall management of a local health facility. The committee is chaired by an elected representative: ward chairperson. Other members include: headteacher of local school, a representative from the local business association, FCHV, ward secretary and a woman nominated by the chairperson. Health facility in-charge acts as a member secretary of the committee [34]	The HFOMC meet once in a month to discuss the health facility operational and management issues. The issues are prioritized, and action plans are made to address the issues. Besides that, HFOMC also involves in dialogue and negotiation in other social accountability interventions, as they are supposed to conduct a social audit in their respective health facility every year. In the other interventions, they are responsible to answer raised concerns and issues [21, 34].	One study [35] reported improved quality of services of birthing centres marked by the availability of 24-hour services, availability of SBA at health facility, improved infection prevention practices and management of labour and delivery in rural health facilities in the areas where HFOMC was actively engaged [35].
ii. **FCHVs:** FCHVs are self-motivated women aged between 25-45 years preferably married, literate and from a disadvantaged group willing to serve the community. They are trained health volunteers responsible to provide promotional maternal and child health services in their community. Some of the FCHVs are also members of HFOMC [29, 36, 37]. Currently, 51,470 FCHVs (47,328 in rural and 4,142 in urban areas) are working across the country [2].	The FCHVs liaison the Mothers’ group and HFOMC in the community level [38]. Moreover, they play an important role in the initial reporting system in community-based MPDSR and voice mechanism of the marginalized and disadvantaged women in the social accountability interventions [2, 21]. Being an important stakeholder of the community, they also monitor and evaluate the performance and quality of health services [36].	No study was found analysing the contribution of FCHV in the health system responsiveness for the quality maternal health services. However, their contribution in the reduction of maternal and child mortality through community-based interventions has been greatly recognized in Nepal [39].
iii. **Mothers’ group for Health**: Mother’s group for health refers to the group of married women belonging to reproductive age (15-49 years) [36].	Every month, the group members meet to discuss various MCH issues and best practices. Similarly, they are responsible to establish and maintain an emergency fund for obstetric services for their fellow group members [36]. They also monitor and evaluate the performances of FCHVs on regular basis and make them accountable towards their roles and responsibilities [21, 36].	A randomized controlled trial (RCT) conducted in a rural Nepal showed that MMR decreased by 80% [adjusted odds ratio (0.22, 95% CI 0.05-0.90)] in the women’s group where community participation project with social accountability interventions was implemented [40]. In the trial groups, the uptake of maternal health services improved and infection prevention practice increased two-fold among the birth attendees [40]. Meanwhile, subgroup analysis of RCTs of the same intervention in four countries, Bangladesh, India, Nepal, and Malawi concluded that at minimum 30% of women participation in the accountability intervention reduced almost half (49%) of the maternal mortality [odd ratio 0.51, 95% CI 0.29-0.89] [41].
iv. **Civil Society Organizations:** The Civil Society Organizations (CSO) refer to the national, local NGOs, international non-governmental organizations (INGOs) and working in the maternal health sector in Nepal [21, 42].	The national NGOs are acting as an oversight mechanism in the health system. They are responsible to facilitate the social audit, CSC/CHSB and community engagement in social accountability interventions. While the INGOs are responsible to provide policy support to the government in the health system strengthening interventions [2, 21].	The CSOs have been recognised for their contribution in health system strengthening and improving maternal and child health outcome through their advocacy role and policy development support to the government [39]. However, no study found exploring the contribution of CSO in the health responsiveness for the quality maternal health services in Nepal and other LMICs.

^a^The word Aama refers Mother in the Nepali language, while “Aama programme” is a maternity scheme introduced under safe motherhood programme to address the financial barriers to access maternal health services by GoN.

### Community engagement

#### Social audit

Social audit is one of the major social accountability interventions which is initiated to improve the transparency of Safe Motherhood Programme and promote equity in the health system. Till fiscal year 2016/17, the programme was scaled up to 70 districts out of 77. The government has a plan to scale up the programme into all 77 districts by 2020 [6, 22].

Given that no scientific research examining the outcomes of social audit in Nepal or any other LMIC was found, the findings from the evaluation and review study of social audit in Nepal has been presented as evidence (Table 5). An evaluation study [23] undertaken in health facilities of Nepal reported overall improvement in the responsiveness of maternal health services. However, the study reported a different level of impact in each study district [22]. It was more effective in Palpa and Rupandehi where the social audit was conducted on regular basis with the technical support of donor agencies compared to Jhapa and Ilam where it was solely implemented by the government without any support from development partners [22]. This result suggests that regular practice of social audit improve maternal health outcomes.

#### Maternal Perinatal Death Surveillance and Response (MPDSR)

WHO has recognized the Maternal Death Review (MDR) as a relevant accountability tool for improved quality of the maternal and neonatal health (MNH) services which are based on the concept of the monitor-review-act cycle [23, 25, 43]. The GoN has merged the MDR program with ‘Maternal Perinatal Death Review (MPDR)’ programme and developed a comprehensive surveillance system named ‘MPDSR’. There are two types of MPDSR: facility-based and community-based in Nepal (see Appendix 3) [23].

The MoHP has established a web-based system to capture maternal deaths in Nepal. With the available data, MoHP has been able to identify the causes of death, and the MPDSR committee has developed action plans for different levels of care; yet, the implementation of those action plans has remained a challenge [2]. Nigeria encountered a similar problem in death reporting and implementation of developed action plans in the initial days. However, they incorporated the scorecard to monitor whether the action plans are being developed and if recommendations were being acted upon accordingly [25]. Based on experiences from Nigeria, this example can possibly be applied in Nepal to overcome the challenges and improve the outcomes of the MDR/MPDSR intervention.

#### Community Score Card/Community Health Score Board (CSC/CHSB)

Both community scorecard (CSC) and community health scoreboard (CHSB) were introduced at the same time in Nepal to promote health sector accountability. The CSC was piloted in 16 health posts by GoN with the support of World Bank in 2011 [26, 27]. CSC was found to contribute to actionable information regarding facility performance; however, due to high implementation cost and lack of competent human resources for the facilitation of the process, the government could not scale it up further in Nepal [21].

Based on the successful experience of CSC in Malawi, CARE-International introduced community health scoreboard (CHSB) in Nepal to improve the maternal health outcomes [28, 30]. Given the contextual similarities between Nepal and Malawi, Nepal is believed to expect similar outcomes of the CSC and/or CHSB in maternal health services. However, the sustainability of the intervention in Nepal is a major challenge, as its execution is being carried out by the donor with minimal involvement of government [21, 44].

#### Citizen Charter

Citizen charter is a tool to ensure the constitutional right of the Nepali citizen to access the information and the commitment of government for providing quality services in a transparent and accountable way [27]. The tool has been used mandatorily in the health sector to ensure transparency, improve service providers’ accountability, make the service users informed about the services and address the concern/grievances of citizen about the services. However, many oversights have been identified while posting information about the availability of essential drugs and services in the health facilities [45]. No evidence is found on the contribution/effectiveness of charter in the maternal health services in Nepal as well as in the other LMICs.

A report about social accountability in the health sector [21] stated that 29% of health facilities do not have citizen charter at their premises and even in places where there are charters, it is not being updated and maintained on a timely basis [21]. This is mainly because of weak enforceability mechanism in the health sector [21, 45].

#### Grievance/complaint handling tool

GoN enforced establishment of a grievance/complain handling mechanism in every public sector including health under the good governance (management and operation) act 2008 [27]. In the health facility, suggestion or complaint boxes are mandatory for handling the grievances and complaints [21]. The box is placed in the premises of health facilities to receive the complaints and/or grievances from the service users, communities as well as other stakeholders about the health services they receive [21].

“… not only at the health facility level but even at the district level, the situation is that suggestion boxes are filled up with ‘spider webs.’ As far as I know the suggestion box is not in use. No one puts their complaints or suggestions [into the suggestion box] by writing onto a piece of paper. Many do not know about its existence. So, I do not see any importance of it”—PHC clinic manager [46].

Moreover, MoHP Nepal has established a digital system to receive complaints via email and Twitter [47]. The district hospitals and district public/health offices are instructed to have their websites which must include information regarding organization, programme and activities including budget and also run a Facebook and Twitter account to foster accountability and transparency and improve service users’ access to the information [21]. Apart from that, MOH has initiated a digital monitoring campaign called ‘Smart Health Nepal’ through its website: https://www.mohp.gov.np [48]. Although the complaint/grievance box contributes to enhancing accountability and transparency, their uses are limited in practice and the required attention is given to systematically place it as a part of a broader accountability system; thus, it fails to serve its purpose [21, 32, 46]. The statement from a study in Nepal as shared in [46] also highlights the attitude of the health workers on the usefulness of complaint box [46].

### Community oversight

#### Health Facility Operation Management Committee (HFOMC)

In line with the objective of Local Self-Governance Act 1999, MoHP devolved its power and responsibilities to HFOMC at the local level for the overall management of their respective health facilities [44, 49]. Furthermore, the local government operation act 2017, has given necessary responsibility to the HFOMC for planning, implementation, and monitoring of the health services [34, 50]. The committee plays an influential role in raising resources for maternal health services in the community. They serve as a strong and inclusive voice mechanism of the community in social accountability interventions [29, 34, 51].

According to the HFOMC guideline, the committee is required to meet once a month to discuss the health facility issues and operational challenges, develop plan of action for effective management of the health facility and review previous action plans, but this is limited in practice [21, 34].

#### Female Community Health Volunteers (FCHVs)

The FCHV programme was initiated in 1988 in Nepal. Initially, the volunteers were assigned to promote Family Planning (FP) services in the community. With improved performance of the programme, their role and responsibilities were gradually expanded to the continuum of care [36]. The FCHVs are known for their remarkable contribution to the reduction of maternal and child morbidity and mortality in Nepal [2, 37].

The FCHVs liaison the mothers’ group and HFOMC at the community level [38]. Moreover, they play an important role in the initial reporting system in community-based MPDSR and voice mechanism of the marginalized and disadvantaged women in the social accountability interventions [2, 21]. Being an important stakeholder of the community, they also monitor and evaluate the performance and quality of health services [36]. Various development partners and government line agencies have mobilized them in health system strengthening programmes recognizing their proximity with the community people and their contribution in MCH sector [21, 39].

#### Mothers’ group for health

The mothers’ group have been recognized as an innovative strategy for ensuring women’s participation to improve the MCH outcomes in Nepal. They are formed at the community level under the initiation of the local health facility [36]. The group is a voice mechanism to raise maternal health concerns in social accountability interventions [21]. This accountability intervention has been considered a cost-effective strategy to save women’s life as per as WHO standard [41]. A study in Nepal concluded that mothers’ group are trusted representatives of women and intermediaries in the social accountability interventions. However, a clear mandate from the policy level is required and their capabilities need to be improved for their active role in social accountability process [29].

#### Civil Society Organization (CSO)

CSOs have been identified as a strong community engagement and oversight mechanism in the accountability and governance process in the health sector [21, 42]. In regards to maternal health, they have played a vital role in advocacy for addressing maternal health problems and promoting accountability from community to central level [39]. In Nepal, local NGOs are mostly involved in implementing social accountability interventions initiated either by the government or development agencies [21]. Some of the NGOs also provide preventive and promotive maternal health services and maternal care too in some cases [2, 21]. Meanwhile, the INGOs provide technical and financial support to the government for the policy and guideline development and for the implementation of social accountability interventions and strategies in the health system [2, 21, 22, 52].

### Contextual factors influencing social accountability interventions in Nepal

#### Social-cultural context

##### Gender Norms

Nepalese society is a patriarchal society where strong gender norms exist. Traditionally, men are privileged with power and position; consequently, women’s participation in the governance system is considerably low [53]. Therefore, to address the issue and empower women for their meaningful participation in each sector of development, GoN has made a reservation for women [42, 54]. Women’s participation has also been ensured in the HFOMC with the mandatory provision of having at least three women out of seven committee members [34].

While government has emphasized women and marginalized groups’ participation in each level of governance to enhance gender equality and social inclusion, they tend to show less interest in accountability interventions due to unequal power relationship, low education, and overload with household chores as well as productive and reproductive works [21, 53, 55]. In most cases, women’s presence in the meetings are not meaningful as they speak only if explicitly requested [53]. Hence women’s roles are confined only up to their physical presence at the programmes while men are the ultimate decision-makers [53]. However, evidence has shown that the increased women’s involvement in participatory decision-making process results in a notable improvement in maternal health services which ultimately reduces maternal mortality and morbidity [40, 41, 56].

##### Social structure

The country has a complex caste system with diverse ethnic groups where the *Brahman*/*Chhetri* refers to the so-called upper caste and are most privileged group while the *Dalit* as disadvantaged caste and *Janjati*—indigenous group—are underprivileged [57]. This caste-based social structure in Nepal has hindered effective participation of the marginalized groups in the social accountability interventions [53]. Earlier, there was a mandatory reservation for a marginalized and disadvantaged group in the HFOMC; in 2019, the provision was abolished from the system. Now, they (one member from *Dalit*, disable and adolescent) are invitee member of the committee, and they do not have an influencing role as a core member in the decision-making process [34].

This caste hierarchy often produces an unequal power relation between the service providers and service users. The marginalized community have less power to negotiate for change in the health service providers attitude and behaviour [31, 59, 60]. In a qualitative study by Gurung et al., a *Dalit* member expressed how caste hierarchy system has suppressed their voice and participation in the accountability process [58].

*“In the committee, most of the members are from higher castes. When we have meetings of the committee or any other programme, and when there is time for taking snacks, the other committee members sit a short distance away from me. There is thus still discrimination in our society. It [untouchability issue] is not in all places, but still exists with some people in some places. Due to this, it causes me stress inside. Then, how can I speak in the meeting or any events without hesitation?”* [58].

Any form of discrimination is prohibited by law in Nepal; yet, the issue of caste-based discrimination is still deeply rooted in the community [54, 58, 61]. Hence, the existing informal power relationship and the dynamics of social structures need to be taken into consideration to ensure the effectiveness of the social accountability process.

##### Awareness, value, beliefs and practices

The effectiveness of social accountability interventions is often influenced by the level of people’s awareness about their rights and entitlements, the existing governance mechanism to protect it and their role in it [12]. In Nepal, majority of the people are unaware of the concept of accountability and governance that makes it difficult to hold service providers accountable for their actions [21, 31]. Although access to the information is a constitutional right of every Nepali citizen, women, the poor and disadvantaged groups are less likely to be aware of their rights to get quality health services [54]. Meanwhile, literacy level, perception and cultural beliefs are hindering factors for it [31, 62].

In Nepal, health belief and practices also influence the level of community participation in social accountability interventions [63]. Similarly, engaging youth and marginalized people in the social accountability interventions are difficult, as youth often hesitate to share their opinion in front of elders and mass, respectively, while marginalized people think their issues are irrelevant to be addressed [63, 64]. Despite having grievances regarding health services and providers’ performance, in most cases, the community tend to stay silent and thus are generally vulnerable and marginalized [46]. Similarly, in Nepalese society, the culture of raising questions and providing feedback to the power holders and prompt respond toward feedback is not properly established which also affect the community engagement in the social accountability interventions [46].

#### Political and economic context

Nepal has gone through various political and structural transitions in the last two decades which has resulted in the unstable political situation and huge governance challenges [31, 44]. Similarly, the issue of political interference in the health sector has been well reported. Often, politics acts as a driving force in the formation and functioning of the HFOMC [58] that interfere with the social accountability process [22, 44]. The decisions are often made on the political ground by the leaders often undermining the community’s concerns. The bureaucracy of health facility, kinship and health worker’s power tends to determine the level of community engagement in the accountability interventions [44, 58, 65].

An evaluation of social audit reported the issue of political pressure in the selection and retention of competent NGO to facilitate the social audit process in the health facilities [22]. Sometimes, intense pressure from government officials and local leaders result in the replacement of experienced NGO with the favoured one which directly affects the quality implementation of social accountability interventions [22]. This kind of political influences tends to increase conflict of interest in social accountability interventions, process and outcomes.

In Nepal, most of the health workers are associated with trade union and sister organization of political parties. The health workers often use this nexus for their deployment and retention at well-facilitated places; it has resulted in a persistent vacant post of regular and skilled staffs in the remote health facilities [44, 65, 66].

#### Health system context

##### Client-provider relationship

Health workers are recognized as an intellectual and respected personality in the community, and their profession is perceived as a highly prestigious profession in Nepal. Hence, the community hardly thinks that health workers commit any mistakes while providing and managing services. This perception often imbalances the power-relation between service users and providers, and often impacts the dialogue and negotiation in the social accountability interventions [46]. In fact, in remote areas where there are no choices for health services, the community often hesitate to complain or to provide feedback to avoid unnecessary conflict and/or the fear of getting poor quality of services in the next visit. A quote from the interview with a NGO staff in a rural health facility reflects the perception of the community toward accountability interventions [46].

*“How can Dalit, women, and the marginalized speak their minds with service providers? They think what the government does is all right. Health is the matter related to life and death. If you or your family member becomes ill, you have to go to the same place. Then, how could you take issue with the service providers? In villages, there is no option”— (Qualitative interview, staff, NGO)* [46].

Sometimes, health workers and HFOMC members also tend to skip the interface with the community due to the fear of being criticized in the accountability interventions like social audit, CHSB etc. [44, 67].

##### Resource availability

Persistent resource deficiency in the health sector limits the health workers’ and policymakers’ responsiveness towards the community [22, 67]. The health facility management has been handed over to the local authority; however, human resource and logistics management are still being performed centrally which has made demand-supply procedure complex [21, 44]. This has resulted in the frequent stock-outs of essential drugs and supplies and scarcity of human resources. Moreover, in the federal government system, the line of accountability between MoHP, provincial and the local government has not been explicitly defined which has further created responsibility dilemma among local authorities to manage health facilities under their responsibilities [21, 44, 61].

The budget provided to local government is insufficient to fulfil the community health priorities identified during the social accountability interventions—a major chunk of it needs to be spent in the staffs’ salary and operation costs [21]. Additionally, due to the human resources gap at community level health facility, FCHVs are overwhelmed with community-based health interventions like Safe Motherhood Programme, FP, immunization, nutrition, MPDSR and mothers’ group meetings, among others. They are also being used by other several sectors such as education, forest groups and micro-credit finance groups for their accountability purpose in the community [37]. Ultimately, they are volunteers and only get an event-based incentive; therefore, increased responsibilities with minimal incentive decrease their motivation to work [36, 37]. This might impact the quality service delivery and their responsiveness toward the community concerns.

##### Monitoring and evaluation

The different levels of GoN monitor social audit process, budget versus expenditure, use of citizen charter, use of grievance handling mechanism, the regularity of HFOMC meeting and regulatory of mother groups meetings. Besides, local councils also monitor programs/activities of the CSOs regularly [44, 50]. However, low responsiveness of the health system has interlinkages with the absence of proper monitoring and evaluation mechanism [6, 49]. Understandably, very few facilities and those located only at feasible geographic locations are frequently monitored and receive regular supervision from the higher authorities [21, 65]. District supervisors are often busy in conducting training and workshops which leave them with less time for supervision and monitoring at community-level health facilities [65]. This has weakened the effectiveness of established social accountability interventions. The regular follow-up and analysis of social audit action plans from the district and/or central level are almost non-existent in the health system [21, 22, 44].

Evidence shows that the demand-side social accountability interventions involve dialogue process which usually puts soft pressure to the health workers to be accountable for their action and responsibility. It is argued that, in the long run, without the threat of penalty from the state or enforcing legal mechanism, the social accountability interventions are likely to address only surface level service delivery issues and thereby affect the sustainability of the interventions [13, 29]. Thus, the health system needs to strengthen the monitoring and supervision mechanism and enforce legal mandate for effective and sustained outcomes of social accountability interventions.

## Discussion

The study found that GoN is committed to improving the responsiveness of maternal health services; hence, various social accountability interventions have been implemented by government/state (supply-side) and development partners (demand-side). Social audit, MPDSR, citizen charter and complaint/grievances handling interventions have been identified as supply-side community engagement interventions, whereas the CSC and CHSB are demand-side interventions. The HFOMC, FCHVs, and mothers’ group for health are a formal structure in the health system to ensure community engagement and oversight in the social accountability interventions. They are considered to bridge the gap between community and health system. Further, they serve as mediators to voice community concerns especially for the women, disadvantaged and marginalized groups in the decision-making process. Meanwhile, CSOs are non-state structures and found to have an advocacy role for the interventions.

This study identified a gap in meaningful community engagement in the accountability process. Mostly, the involvement of women, Dalit and adolescents in the HFOMC meetings and the decision-making process is often done as a formal procedure. Similarly, their level and depth of participation have not been utilized properly [21, 46, 53, 58]. In brief, strong gender norms, caste discrimination, political and economic influences, resource deficiencies and weak enforceability mechanism are found as the most influencing factors for social accountability interventions in Nepal which need to be considered while designing and implementing the interventions.

Social audit and MPDSR are widely promoted supply-side interventions in maternal health services by GoN. This review showed that the government has contributed to improving the responsiveness of Nepal’s health system and the delivery of quality maternal health services. However, further scientific studies are needed to generate a strong argument for that. Despite its effectiveness, there is a gap in the quality implementation of the social audit which consequently affects its outcomes. This is due to the contextual factors such as weak monitoring and regulatory mechanism, political interference, resource constraints in the health system and power limitation at the local level. The social audit has been practised as an event to expenses budget rather than a process; thus, interventions are not producing expected outcomes. There is a lack of reflection on the processes and built action plans from related higher authorities. Additionally, a gap identified in the integration of developed action plans during social audit into the policy level decision-making aggravates the problem further [21, 22, 44].

Concerning MPDSR, further research is recommended to generate strong evidence. MPDSR could be effective to improve the quality of maternal health services and avoid ‘delays’ in receiving the services if the developed action plans are implemented [10, 24, 25, 43]. However, as the tool specifically focusses on individual mistakes, it might promote fear among health care workers around the management of obstetric emergency care. Therefore, it is imperative to consider constructive environment focuses on systematic weakness with an opportunity to improve [25].

Considering the difficulties of illiterate people who cannot read the information written in citizen charter, it is necessary to prepare alternatives to inform people about the availability of services. Disseminating the information from television, radio and mobile phone can be alternative means for these groups. The fact that almost every household in Nepal has at least one mobile phone [5] shows the opportunity of information dissemination via short message or voice message. Though the short message may not work for illiterate people, voice message or toll-free call services can be considered more effective message dissemination tool for such communities.

A report by Commissions for the Investigation of Abuse of Authority (CIAA) showed the health sector in Nepal in 5th place amongst the 9 sectors for the highest number of complaint about power abuse which indicated potential corruption vulnerabilities in the health system [68]. To address such serious issue, CSOs and local government need to collaborate with the local councils for enforcement of regulations for the internal control systems of the local governance ordinance [21].

Mafuta, in his research [12], highlighted that social accountability interventions work effectively in maternal health services if women are allowed to express their concerns and have access to channels to provide feedback, and health service providers are positive toward user feedback and willing for behavioural changes [12]. This is relevant to Nepal as well as past researches which have shown a positive correlation between women participation in social accountability intervention and its impact on maternal health outcomes [40, 41].

Poor information dissemination might result in information asymmetry between providers and users, ultimately affecting the quality of the service delivery. Without awareness, the community may not be able to evaluate the components of QoC in maternal health services and to claim their rights entitlements; this ultimately leads to superficial community participation and produce suboptimal outcomes [69]. The study also identified a gap in the complaint capturing and handling mechanism in the health system. Community people often prefer informal channels to express their complaint/grievances or provide feedback in Nepal. The initiatives of MoHP through a web-based complaint handling mechanism [48], however, may not be feasible for the local population who are not internet users and technology-friendly. According to the International Telecommunication Union (ITU), only 34% of the Nepalese population has access to the internet [70]. Therefore, an effort is needed to establish other informal channels such as mobilization of committee members, mothers’ group and anonymous complaint registry through call, among others, to acquire the relevant feedback.

Political instability and bureaucratic influences have resulted in low trust among the community over the state, and thus, the government needs to put an effort in improving trust between citizen and state [13, 44, 65]. This will help to improve the client-provider relationships and effectiveness of social accountability interventions. To do so, it is very necessary to improve access to information for the community and increase their awareness of the right entitlements and legal enforcement.

Without sufficient resources and power at the local level, social accountability cannot produce optimal outcomes. Studies have shown that fulfilment of the permanent post, construction of major infrastructures, purchase of drugs and commodities and improving monitoring and a supervisory visit to health facilities, etc are not under the control of health management committee and community people [21, 22, 30, 44, 58]. Therefore, there is a need for proper devolution of power at a local level and strengthening of their political capabilities. This would be a critical step to avoid political and economic interference and power symmetry as well as contribute to managing resource constraints at the local level.

For the evidences to drive into action in maternal health services, they need to be designed and communicated in a way that triggers the policymakers’ will to address persistent resource scarcity [71]. Local NGOs and INGOs can play a crucial role to enforce policy into action as they are mostly engaged in an advocacy role and policy support at multiple levels.

Regarding demand-side interventions, the study found that they have proven to improve the quality of maternal health services in different LMICs including Nepal. Nevertheless, despite their effectiveness of improving health system responsiveness, some limitations have been observed. For example, CHSB is focused on specific groups and is project-oriented where sustainability largely depends on the donors funding. Similarly, MOHP is not able to scale-up CSC due to resource constraint and lack of technical competencies. The study found that donor-initiated interventions have been implemented without a clear vision of their sustainability [21, 44]. The CSC, CHSB and social audit have many similarities; thus, implementation of fragmented interventions may lead to duplication of efforts and wastage of resources. Therefore, specific and collaborative effort is required from both government and development partners for the integration of those interventions and institutionalization of those interventions to improve efficiency and to ensure the sustainability which will ultimately improve maternal health services in Nepal.

## Limitations

The findings of this study ground on literature review and desk study of the available scholarly literature and study reports. Among them, very limited researches provided a comprehensive information on the outcomes of social accountability interventions in the maternal health services in Nepal. This might be due to the lack of required emphasis on the focused studies. Given this condition, the studies from LMICs having similar maternal health and socio-economic context are presented to give an overview of how social accountability improves maternal health services in the contexts like Nepal.

Furthermore, the analysis of the study focused on quality issues in public health facilities. Understandably, private health institutions providing maternal health services also have cases of poor QoC, but they have not been explored in this review [6, 8]. Conclusions, therefore, should be comprehended and interpreted considering this limitation as well. None of these limitations, however, should be understood as a compromise to the quality issues; they have been ensured by reviewing all peer-reviewed articles, national-level survey reports and evaluation studies, and evidences published in official governmental and organizational websites.

## Conclusions

Various social accountability interventions, both at supply-side and demand-side, have been implemented to improve the responsiveness of maternal health services in Nepal. Despite the effective outcomes of the supply-side interventions, quality implementation is a key issue. Insufficient monitoring and evaluation, resource constraints, weak response system and political influences are identified as major responsible factors. Demand-side interventions are also found to have positive influences on the health system responsiveness. However, the interventions are project-oriented and donor-dependent. Therefore, clear policy guidance is needed for standardization and sustainability of the donor-initiated social accountability interventions. Similarly, contextual factors, such as socio-cultural factors (gender norms, social structure, value beliefs), health system factors (client-provider relationship, resource availability, monitoring and evaluation), and political-economic factors are found to operate as determining factors for effectiveness and sustainability of the social accountability interventions. Eventually, the evidence that directly links social accountability to improved maternal health services is not strong. Therefore, there is a strong need for investment in research in social accountability interventions and its impact on maternal health services in Nepal to enhance evidence-based practices and approaches.

## Supplementary Information

**Additional file 1: Appendix 1.** Search strategy

**Additional file 2: Appendix 2.** References details

**Additional file 3: Appendix 3.** Flow diagram of MPDR and MPDSR [23]

## Data Availability

Not applicable

## Abbreviations

ANC: Antenatal care
CARE: Cooperative for Assistance and Relief Everywhere
CHSB: Community Health Score Board
CSC: Community Score Card
CSOs: Civil Society Organizations
DFID: Department for International Development
DHO/DPHO: District Health Office/District Public Health Office
DoHS: Department of Health Services
FCHVs: Female Community Health Volunteers
HFOMC: Health Facility Operation and Management Committee
INGOs: International Non-Governmental Organizations
LMICs: Low and middle-income countries
MCH: Maternal and child health
MDR: Maternal death review
MMR: Maternal mortality ratio
MNH: Maternal and neonatal health
MoFALD: Ministry of Federal Affairs and Local Development
MoH: Ministry of Health
MoHP: Ministry of Health and Population
MPDR: Maternal Perinatal Death Review
MPDSR: Maternal Perinatal Death Surveillance and Response
NHRC: Nepal Health Research Council
NHSS: Nepal Health Sector Strategy
PHC: Primary health care
QoC: Quality of care
RCT: Randomized controlled trial
SDGs: Sustainable development goals
SBA: Skilled birth attendant
UHC: Universal Health Coverage
WHO: World Health Organization
