# Supplementary figures and images for: Improving maternal health services through social accountability interventions in Nepal: an analytical review of existing literature
Source: Public Health Rev. 2020 Dec 21;41:31. doi: 10.1186/s40985-020-00147-0 (PMC7751117; doi:10.1186/s40985-020-00147-0)

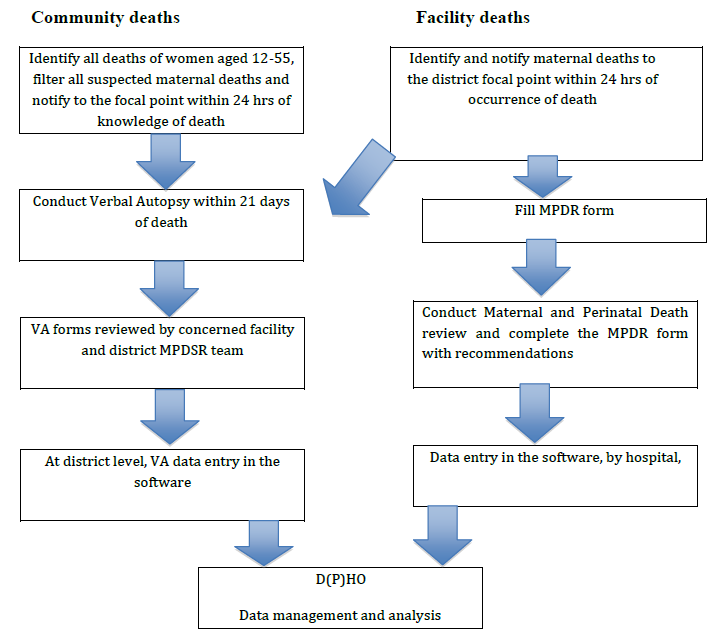


Appendix 3: Flow diagram of MPDR and MPDSR [23].

Supplement: Supplementary file 3 — Additional file 3: Appendix 3. Flow diagram of MPDR and MPDSR [23] [file 40985_2020_147_MOESM3_ESM.docx]
